# Treatment With Tetrahydrobiopterin Overcomes Brain Death–Associated Injury in a Murine Model of Pancreas Transplantation

**DOI:** 10.1111/ajt.13364

**Published:** 2015-06-23

**Authors:** R. Oberhuber, P. Ritschl, C. Fabritius, A.‐V. Nguyen, M. Hermann, P. Obrist, E. R. Werner, M. Maglione, B. Flörchinger, S. Ebner, T. Resch, J. Pratschke, K. Kotsch

**Affiliations:** ^1^ Center for Operative MedicineDepartment of VisceralTransplantation and Thoracic Surgery, Innsbruck Medical UniversityInnsbruckAustria; ^2^ Department of Anaesthesiology and Critical Care MedicineInnsbruck Medical UniversityInnsbruckAustria; ^3^ Department of PathologySt. Vincent's HospitalZamsInnsbruckAustria; ^4^ Division of Biological Chemistry, BiocenterInnsbruck Medical UniversityInnsbruckAustria; ^5^ Department of Cardiothoracic SurgeryRegensburg University HospitalRegensburgGermany; ^6^ Department of Visceral, Abdominal and Transplantation SurgeryCharité‐UniversitätsmedizinBerlinGermany

**Keywords:** donors and donation: donation after brain death (DBD), ischemia reperfusion injury (IRI)

## Abstract

Brain death (BD) has been associated with an immunological priming of donor organs and is thought to exacerbate ischemia reperfusion injury (IRI). Recently, we showed that the essential nitric oxide synthase co‐factor tetrahydrobiopterin (BH4) abrogates IRI following experimental pancreas transplantation. We therefore studied the effects of BD in a murine model of syngeneic pancreas transplantation and tested the therapeutic potential of BH4 treatment. Compared with sham‐operated controls, donor BD resulted in intragraft inflammation reflected by induced IL‐1ß, IL‐6, VCAM‐1, and P‐selectin mRNA expression levels and impaired microcirculation after reperfusion (p < 0.05), whereas pretreatment of the BD donor with BH4 significantly improved microcirculation after reperfusion (p < 0.05). Moreover, BD had a devastating impact on cell viability, whereas BH4‐treated grafts showed a significantly higher percentage of viable cells (p < 0.001). Early parenchymal damage in pancreatic grafts was significantly more pronounced in organs from BD donors than from sham or non‐BD donors (p < 0.05), but BH4 pretreatment significantly ameliorated necrotic lesions in BD organs (p < 0.05). Pretreatment of the BD donor with BH4 resulted in significant recipient survival (p < 0.05). Our data provide novel insights into the impact of BD on pancreatic isografts, further demonstrating the potential of donor pretreatment strategies including BH4 for preventing BD‐associated injury after transplantation.

## Abstract 2

In a syngeneic model of pancreas transplantation, the authors illustrate that donor brain death results in intragraft inflammation and impairs microcirculation, whereas pretreatment of the brain‐dead donor with BH4 significantly improves microcirculation and cell viability.

**Abbreviations**
BD: brain dead
BH4: tetrahydrobiopterin
CD: capillary diameter
DCD: donation after circulatory death
DGF: delayed graft function
FCD: functional capillary density
IRI: ischemia reperfusion injury
NOS: nitric oxide synthase

## Introduction

Simultaneous kidney–pancreas transplantation is the therapy of choice for selected patients suffering from diabetes mellitus and end‐stage renal disease 1, 2, 3. Despite various improvements in surgical techniques, organ procurement, preservation, and novel immunosuppressive regimens, there remains an ever‐increasing disparity between patients on waiting lists and available organs for transplantation. For instance, the percentage of patients on the pancreas waiting list increased by more than 150% over the last decade, while the number of pancreas transplantations performed in the same period increased by only 21%. In general, the majority of pancreata are procured from donors diagnosed as brain dead (BD) 1. Moreover, the incidence of organs derived from nonstandard deceased donors, including pediatric deceased donors, organs from aged or overweight BD donors is increasing and has been associated with poorer short‐ and long‐term graft survival 3, 4. In order to address the increasing gap between supply and demand of suitable organs numerous efforts to increase the donor pool are currently under consideration. These include the use of pancreata procured through donation after circulatory death (DCD) 5. Although outcomes for kidney and liver grafts from DCD donors have become increasingly successful, experience with pancreata procured from these donors is still limited 6.

Importantly, outcomes of kidneys from BD donors are still significantly inferior to those from living donors, a fact that cannot be fully attributed to shorter cold ischemia time or better HLA matching. There is a growing body of evidence to show that non‐allo‐immunological factors including BD and ischemia reperfusion injury (IRI) play a crucial role in impaired short‐ but, even more importantly, in long‐term graft survival rates 7, 8, 9. The occurrence of BD is linked to hemodynamic fluctuations, organ hypoperfusion, hypothermia, coagulopathy, hormone depletion, and electrolyte abnormalities 10. In this context, donor BD has been shown to provoke increased expression of pro‐inflammatory cytokines, endothelial activation, increased expression of MHC class II molecules, infiltration of the donor organ with immune cells, and activation of the complement system 11. This inflammation is further exacerbated by ischemia and, paradoxically, by the reinstitution of blood supply during organ reperfusion 12. Consequently, the combination of donor BD and IRI causes enhanced immunogenicity of the graft, which accelerates the recipient's immune response after transplantation. This is clinically reflected by a higher incidence of delayed graft function (DGF) and impaired long‐term outcome in kidney transplantation 7, 13, 14 and even more in pancreas transplantation, IRI‐associated pancreatitis with subsequent pro‐thrombogenicity is one of the leading causes of early graft failure 2.

Tetrahydrobiopterin (BH4), an essential nitric oxide synthase (NOS) cofactor, exerts profound effects on the structure of all NOS isoforms by stabilizing the active homodimeric form of the enzyme and increasing substrate affinity 15. Oxidative stress, for example, during IRI has been shown to deplete intracellular BH4 stores below a critical threshold, causing the so‐called “uncoupling” of the NOS enzyme, which produces superoxide anions and hydrogen peroxide instead of NO, finally causing tissue damage 16, 17. We have already shown that following murine pancreas transplantation IRI was effectively prevented by donor pretreatment with BH4 after prolonged cold ischemia time of 16 h and that this protective effect of BH4 treatment is NOS‐specific 18, 19, 20, 21. Therefore, the aim of our study was to investigate the impact of donor BD in a murine model of syngeneic pancreas transplantation in order to elicit the therapeutic potential of BH4 in reducing BD‐ and IRI‐related tissue damage after transplantation.

## Research Design and Methods

For real‐time RT‐PCR, PCR primers, serum amylase, and lipase measurements, determination of BH4 tissue levels, histopathology, and immunohistochemistry, please refer to the Supporting Information.

### Animals

Male C57BL/6 (H‐2^d^) mice aged 8–12 weeks were purchased from Charles River (Sulzfeld, Germany). Animals were housed under standard conditions and received humane care in compliance with the “Principles of Laboratory Animal Care” prepared by the National Academy of Sciences and published by the National Institutes of Health (NIH Publication No. 86‐23, revised 1985). All experiments were approved by the Austrian Ministry of Education, Science and Culture (BMWF‐66.011/0144‐II/10b/2012).

### Experimental design

The experiment design consisted of eight groups (n = 5–8 animals/group): Group I: untreated nontransplanted group (“naïve”); Group II: nontransplanted sham group (“sham only”); Group III: nontransplanted BD group (“BD only”); Group IV: nontransplanted BD group treated with BH4 (“BD only plus BH4”); Group V: untreated transplanted group (“Tx non‐BD”); Group VI: untreated transplanted sham group (“Tx sham”); Group VII: transplanted BD group untreated (“Tx BD”); Group VIII: transplanted BD group treated with BH4 (“Tx BD plus BH4”). Moreover, four groups (n = 5) were used for survival analysis. Group IX: untreated transplanted group (“Tx non‐BD”); Group X: untreated transplanted sham group (“Tx sham”); Group XI: transplanted BD group untreated (“Tx BD”); Group XII: transplanted BD group treated with BH4 (“Tx BD plus BH4”). Observation period consisted of 20 days, providing an independent solid readout for graft outcome. For graft recovery, Custodiol perfusion solution (HTK, Dr. Franz Köhler Chemie GmbH, Alsbach‐Hähnlein, Germany) was used. BH4 [(6R)‐5,6,7,8‐tetrahydro‐l‐biopterin dihydrochloride] (Schircks Laboratories, Jona, Switzerland) treatment of graft donors consisted of a single dose of 50 mg/kg b.w. 2 min before pancreas recovery or assessment. No treatment was applied to recipients. I.m. administration was chosen because of its suitability in this model and because of the previously shown rapid uptake of pteridines 19, 22.

### Murine model of brain death induction

After a cervical midline skin incision a blunt‐tipped 26G cannula was inserted into the right common carotid artery and connected to a transducer for continuous blood pressure monitoring (Biopac Systems Inc., Santa Barbara, CA). Next, a tracheostomy was performed to insert a ventilation cannula that was connected to a ventilator (Harvard Apparatus Inc., Holliston, MA). Ventilation of the animal was then started at a respiration rate of 150 min and a tidal volume of 200 µL. Trepanation was performed and a Fogarty catheter (No. 3, Fogarty Arterial Embolectomy Catheter, Baxter Healthcare, Unterschleissheim, Germany) was inserted in the epidural space. For BD induction, the catheter was inflated with saline over a period of 15 min to achieve brain stem compression and irreversible pontine ischemia. BD was clinically documented as a characteristic initial blood pressure peak, subsequent transient spontaneous muscular fasciculations of the rear limbs, and the absence of spinal reflexes. Saline was used for volume resuscitation. Animals with refractory hypotensive episodes (MAP<55 mmHg) of more than 10 min duration were excluded from the experiments. After BD induction animals were ventilated for 3 h before organ retrieval. Sham animals (Groups III, VII, and X) underwent the same procedure (blood pressure monitoring, ventilation time, same amount of saline injection, and trepanation) without BD induction (the catheter was placed in the epidural space, however, it was not inflated) 23. Naïve animals were anesthetized and sacrificed thereafter. After BD was induced, decreasing blood pressure levels became evident over the 3 h observation period. However, mean blood pressure levels at defined time points (i.e. 30, 60, 90, 120, 150, and 180 min after BD induction) were comparable in BD animals, sham controls, and BD+BH4‐pretreated animals (Figure S1).

### Pancreas transplantation

Animals were anesthetized with an intramuscular injection of 100 mg/kg b.w. ketamine hydrochloride (Ketavet, Pharmacia GmbH, Erlangen, Germany) and 10–15 mg/kg b.w. xylazine (Xylasol, Dr. E. Gräub AG, Bern, Switzerland). Through a midline abdominal incision pancreatic grafts with the spleen were explanted by sequential separation of the duodenum and the mesenteric axis. Exocrine secretion was managed by ligation of the choledocho‐pancreatic duct. Consequently, no exocrine drainage was necessary. After 45 min of cold ischemia, pancreatic grafts were placed in the cervical region of the recipient and revascularized by anastomosis with the recipient's common carotid artery and external jugular vein using a modified non‐suture cuff technique. Prior to reperfusion the spleen was removed 24.

### Confocal intravital fluorescence microscopy

Confocal intravital fluorescence microscopy (IVFM) was performed using a microlens‐enhanced Nipkow disk‐based confocal system UltraVIEW RS (Perkin Elmer, Wellesley, MA) mounted on the Olympus IX‐70 inverse microscope (Olympus, Nagano, Japan) at two different times: before organ retrieval (nontransplanted groups; I–IV), or 2 h following organ reperfusion (transplanted groups; V–VIII). Microvascular disorders were quantified by mean functional capillary density (FCD), defined as the length of all blood cell‐perfused nutritive capillaries per observation area and by the mean capillary diameter (CD), defined as the mean of the three largest capillaries per observation area. In order to enhance microvessel contrast, 0.3 mL of a 0.4% fluorescein‐isothiocyanate‐labeled dextran (Sigma–Aldrich, Vienna, Austria) was injected into the animals' penile vein. For assessment of cell viability of pancreatic tissue, biopsies were placed in 200 µL HEPES‐buffered RPMI (Sigma, Vienna, Austria) medium at room temperature in 8‐well‐chambered cover glasses (Nalgene Nunc. International, Rochester, NY). After adding corresponding live stains, samples were incubated for 15 min. SYTO®16 in combination with propidium iodide (PI) was used to assess and quantify viable and nonviable cells within the biopsies 25.

### Statistics

Results are expressed as mean ± standard error of the mean (SEM). Statistical analysis was performed using GraphPad Prism 5 (GraphPad Software, La Jolla, CA). One‐way ANOVA for normally distributed data was calculated for kinetic studies. Adjustments for multiple comparisons were performed with the Tukey–Kramer Multiple Comparisons Test. Kaplan–Meier curve was used for survival analyses and survival differences between groups were compared using the log rank test. A p value of <0.05 was considered statistically significant.

## Results

### Intragraft inflammation from donor BD is ameliorated by BH4 pretreatment

**Figure 1:**
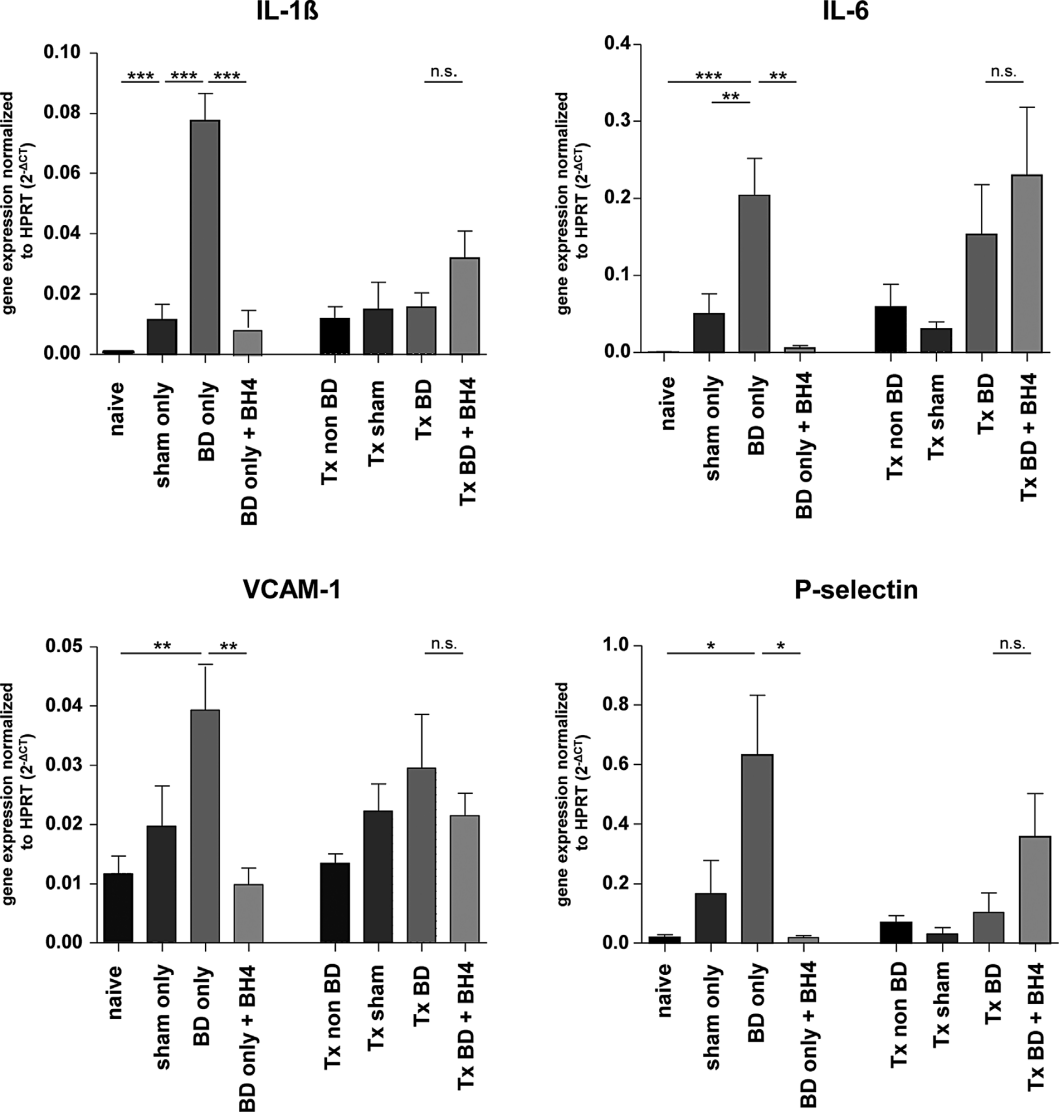
**Donor BD significantly induces inflammatory candidate markers in the pancreas** mRNA expression levels of IL‐1ß, IL‐6, VCAM‐1, and P‐selectin in pancreatic grafts. Significant changes in IL‐1ß and IL‐6 were observed for BD only grafts in comparison to sham and naïve animals (p < 0.001 and p < 0.01, respectively). Although both adhesion molecules VCAM‐1 and P‐selectin were clearly induced, statistical significance was reached only for BD only versus naïve animals (p < 0.01 and p < 0.05, respectively). No significant changes in gene expression were detected in pancreatic grafts derived from the transplanted group. Data are presented as mean values of n = 6–8 animals/group ± SEM. Statistically significant differences were tested with one‐way ANOVA and the Tukey posttest; *p < 0.05, **p < 0.01, ***p < 0.001. ANOVA, analysis of variance; BD, brain dead; BH4, tetrahydrobiopterin; HPRT, hypoxanthine hosphoribosyltransferase; Tx, transplantation.

In order to detect intragraft inflammation following BD and IRI, we assessed the inflammatory profile of pancreata by measuring candidate genes indicative for inflammation and adhesion. Interestingly, we detected a clear induction of all four candidate genes including IL‐1ß, IL‐6, VCAM‐1, and P‐selectin as a consequence of donor BD only. However, solely data for IL‐1ß and IL‐6 mRNA expression were significantly enhanced as compared to sham controls (BD only vs. sham only; p < 0.001, p < 0.01) (Figure 1) whereas pretreatment of the BD donor with BH4 resulted in significant downmodulation for all investigated genes (p < 0.05, p < 0.01, and p < 0.001). Contrarily, no significant changes as a consequence of BD and IRI were detected (Figure 1).

### Donor BD in combination with IRI impairs pancreatic microcirculation

**Figure 2:**
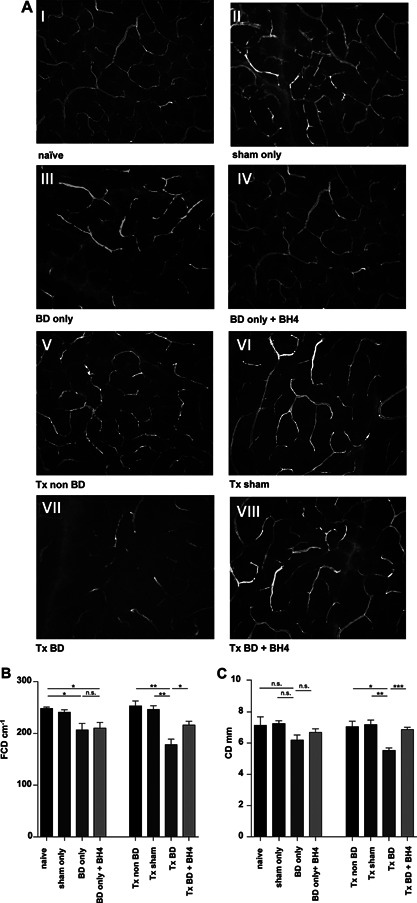
**Pancreatic microcirculation is disturbed following BD and IRI, but significantly improved following BH4 treatment** (A) Fluorescein‐labeled dextran was used to visualize the capillary mesh of nontransplanted organs from: naïve animals (I), sham‐treated animals (II), BD donors (III), and BD donors treated with BH4 (IV). After syngeneic transplantation of pancreata derived from non‐BD donors into recipients an almost physiological capillary mesh was detected (V). Sham‐treated grafts displayed a regular capillary mesh after transplantation (VI). Severe microcirculatory alterations were detected after transplantation of untreated pancreata from BD donors (VII). In contrast, an amelioration of capillary graft microperfusion in the BH4 treatment group was observed (VIII). Representative pictures are shown for n = 5 animals/group. Observation area: 0.00159 cm^2^ (454 µm × 349 µm). (B) FCD defined as the length of all blood cell‐perfused capillaries per observation area. (C) CD defined as the mean of the three largest capillaries per observation area. Data are presented as mean of n = 5 animals/group ± SEM. Statistically significant differences were tested with one‐way ANOVA and the Tukey posttest; *p < 0.05, **p < 0.01, ***p < 0.001. ANOVA, analysis of variance; BD, brain dead; BH4, tetrahydrobiopterin; CD, capillary diameter; FCD, functional capillary density; IRI, ischemia reperfusion injury; SEM, standard error of the mean; Tx, transplantation.

As illustrated by representative IVFM microscopic pictures of nontransplanted pancreatic tissues (Figure 2A, Groups I–IV), donor BD (BD only) clearly disturbed pancreatic microcirculation, while BH4 treatment (BD only + BH4) demonstrated only a limited effect. In contrast, the combination of BD and IRI produced a marked breakdown in microcirculation of untreated, transplanted animals (Tx BD; Figure 2A Group VII), whereas BH4‐treated grafts displayed a regular capillary mesh comparable to the Tx sham and Tx non‐BD groups (Figure 2A, Group VIII vs. Groups V and VI). The extent of microvascular injury was further assessed by measuring functional capillary density (FCD). Donor BD resulted in significantly lower FCD than in naïve animals (BD only vs. naïve; 206.6 cm^−1^ ± 12.5 vs. 248.0 cm^−1^ ± 3.130; p < 0.05). However, BH4 treatment had no significant impact on pancreatic microcirculation prior to transplantation (BD only vs. BD only + BH4, 206.6 cm^−1^ ± 12.5 cm^−1^ vs. 210.2 ± 11.03; p = n.s.) (Figure 2B). Contrarily, donor BD resulted in significantly lower FCD values than in grafts from the transplanted sham group (Tx BD vs. Tx sham; 178.4 cm^−1^ ± 10.67 vs. 246.3 cm^−1^ ± 7.197; p < 0.01) or the transplanted non‐BD group (253.1 cm^−1^ ± 9.733; p < 0.01). Importantly, administration of BH4 resulted in significantly increased FCD as compared to nontreated grafts (Tx BD plus BH4 vs. Tx BD; 216. 1 cm^−1^ ± 7.25; p < 0.05) (Figure 2B).

Next, vascular‐mesh irregularities in pancreatic tissue were quantified in terms of capillary diameter (CD). Mean CD results followed patterns similar to those for FCD, as CD values were comparable in nontransplanted groups because no statistically significant influence of BD only or of BH4 treatment was detected (Figure 2C). However, transplantation of pancreata from BD donors resulted in significantly lower CD than for grafts from the transplanted sham group (Tx BD vs. Tx sham; 5.54 ± 0.16 vs. 7.18 ± 0.29; p <0.01) or the non‐BD group (Tx BD vs. Tx non BD, 7.06 ± 0.34; p < 0.05). Again, donor treatment with BH4 resulted in significantly increased mean CD as compared to nontreated grafts (Tx BD plus BH4; 6.87 ± 0.14; p < 0.001) (Figure 2C).

### Donor BD in combination with IRI dramatically impacts cell viability

**Figure 3:**
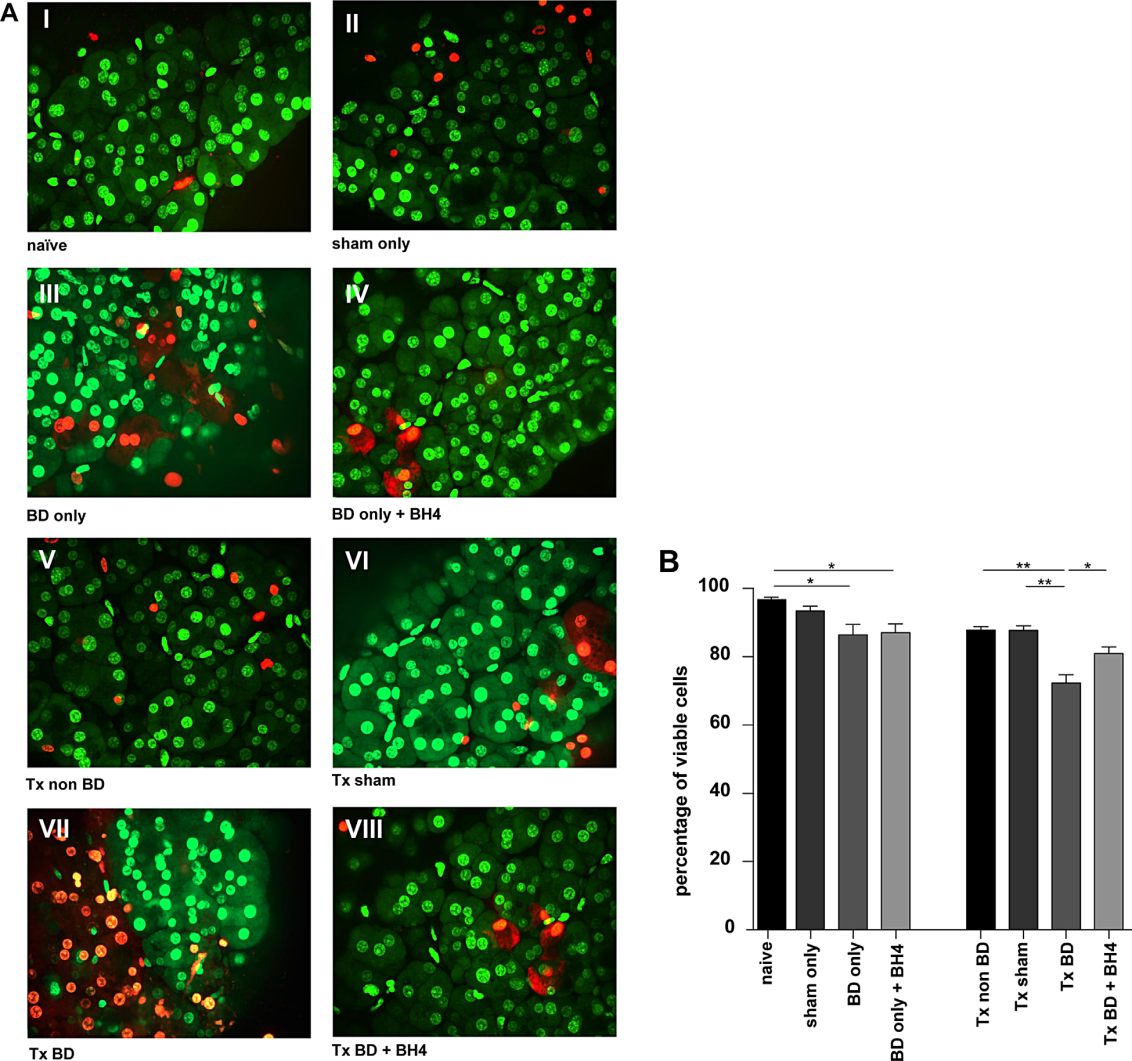
**BD and IRI impair cell viability of pancreatic isografts, which is ameliorated by BH4 treatment of the donor** (A) Representative pictures of nontransplanted organs derived from naïve controls (I); sham‐treated animals (II); BD donors (III), BD donors treated with BH4 (IV). Grafts from non‐BD donors showed high cell viability after transplantation (V), similar to sham‐treated donors (VI). Transplantation of pancreata from BD donors into syngeneic recipients resulted in markedly reduced cell viability (VII), whereas amelioration of cell viability was observed in the treatment group (VIII). Representative pictures are shown for n = 5 animals/group. (B) The percentage of viable cells in biopsies taken after organ procurement or 2 h after reperfusion is shown. Data are presented as mean values of n = 5 animals/group ± SEM. Statistically significant differences were tested with one‐way ANOVA and the Tukey posttest; *p < 0.05, **p < 0.01. ANOVA, analysis of variance; BD, brain dead; BH4, tetrahydrobiopterin; IRI, ischemia reperfusion injury; SEM, standard error of the mean; Tx, transplantation.

In alignment with the results derived from the microcirculation assessment (Figure 2A–C), viability analysis revealed some impact of BD, assessed in pancreatic biopsies taken 3 h after BD induction (Figure 3A, Groups I–IV). Donor BD resulted in a significantly poorer cell viability than in naïve animals (BD only vs. naïve; 86.47 ± 2.98 vs. 96.77 ± 0.54; p < 0.05). However, BH4 treatment exerted no significant impact on cell viability prior to transplantation (BD only vs. BD only plus BH4, 86.47 ± 2.98 vs. 87.04 ± 2.61; p = n.s.) (Figure 3B). In contrast, the combination of BD and IRI had a dramatic impact on cell viability (Figure 3A, Groups V–VIII). Grafts from BD donors displayed significantly poorer cell viability than did grafts from the transplanted sham group (Tx BD vs. Tx sham; 72.27% ± 2.42 vs. 87.68% ± 1.4; p < 0.01) or the non‐BD group (Tx BD vs. Tx non BD 87.77% ± 1.12; p < 0.01). Treatment with BH4 resulted in statistically significantly better cell viability of grafts from BD donors than of nontreated grafts (Tx BD plus BH4 vs. Tx BD; 80.84 ± 2.00 vs. 72.27% ± 2.42; p < 0.05) (Figure 3B).

### Treatment with BH4 ameliorates parenchymal damage of pancreatic tissue after BD and IRI

**Figure 4:**
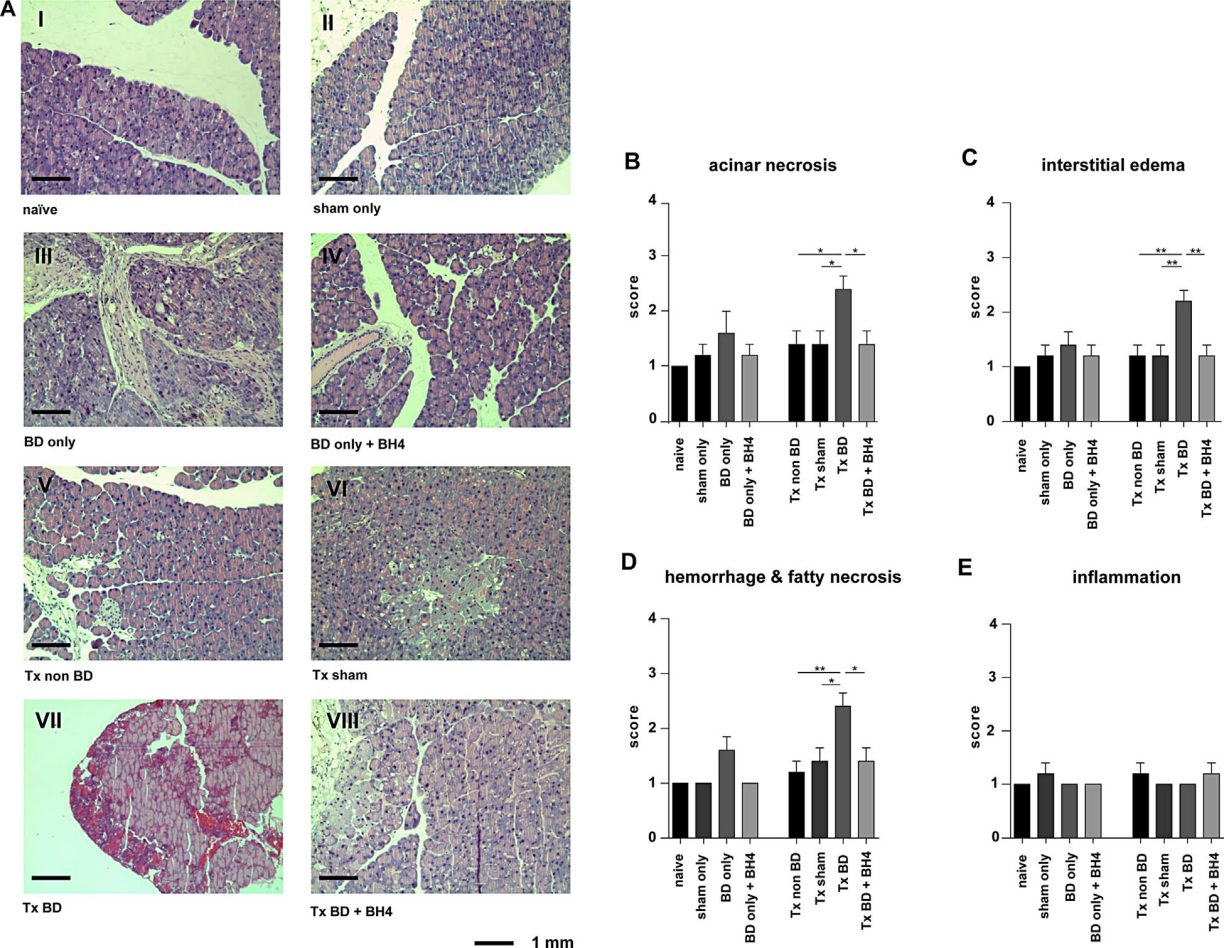
**BH4 pretreatment results in improved graft histology reflected by reduced levels of necrosis, edema, hemorrhage, and fatty necrosis** (A) Histological evaluation of nontransplanted pancreatic grafts from naïve animals (I), or sham‐treated animals (II) showed no signs of parenchymal damage. Analysis of pancreatic tissue from BD donors (III) revealed some impact of BD on parenchymal damage as reflected by signs of interstitial edema, acinar vacuolization, infiltration with few inflammatory cells, and necrosis of the adjacent fat tissue. Treatment using BH4 displayed some effect (IV). In contrast, the combination of donor BD and ischemia reperfusion severely damaged graft parenchyma characterized by massive hemorrhages and fat necrosis, cell swelling and edema, and necrosis of the acinar cells (VII), whereas treatment with BH4 protected grafts from tissue damage (VIII). Grafts from sham‐treated (VI) and non‐BD (V) control animals showed a slight edema 2 h after transplantation. Representative pictures are shown for n = 5 animals/group. (B–E) Graphs reporting the semiquantitative histopathological Schmidt score including (B) acinar necrosis, (C) interstitial edema, (D) hemorrhagic and fat necrosis, and (E) inflammatory infiltrates. Data are presented as mean values of n = 5 animals/group ± SEM, and statistically significant differences were tested with one‐way ANOVA and the Tukey posttest; *p < 0.05, **p < 0.01. ANOVA, analysis of variance; BD, brain dead; BH4, tetrahydrobiopterin; SEM, standard error of the mean; Tx, transplantation.

Histomorphological analysis revealed only a marginal impact of BD or BH4 treatment on nontransplanted groups (Figure 4A, Groups I–IV), and none of the four subcategories of the Schmidt score achieved statistically significant differences (Figure 4B–E). In contrast, transplantation of grafts from BD donors resulted in severe parenchymal damage following 2 h of reperfusion with significantly higher scores for acinar necrosis (p < 0.05), interstitial edema (p < 0.01), hemorrhagic or fat necrosis (p < 0.05, Figure 4A–D) than for sham controls. No statistically significant differences were detected with respect to the extent of infiltration of inflammatory cells (Figure 4E). In addition, BH4 treatment ameliorated tissue damage as displayed by significantly lower mean scores for acinar necrosis (p < 0.05), interstitial edema (p < 0.01), hemorrhagic, or fat necrosis (p < 0.05, Figure 4A–D) than in nontreated BD animals.

### Donor BD and IRI accelerate pancreatitis

**Figure 5:**
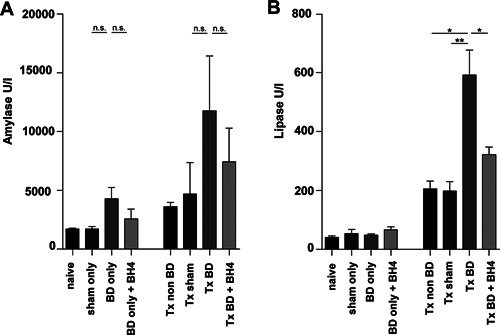
**Reduced pancreatic lipase levels following BH4 pretreatment** (A) While donor treatment using BH4 resulted in markedly reduced amylase levels in isograft recipients, no statistical significance was observed (Tx BD vs. Tx BD plus BH4, p = n.s.). (B) In contrast, lipase levels in the same group were significantly higher than in BH4‐treated donors. Data are presented as mean values of n = 5 animals/group ± SEM and statistically significant differences were tested with one‐way ANOVA and the Tukey posttest; *p < 0.05, **p < 0.01. ANOVA, analysis of variance; BD, brain dead; BH4, tetrahydrobiopterin; SEM, standard error of the mean; Tx, transplantation.

In order to assess the grade of pancreatitis clinically relevant markers including amylase and lipase were determined in the recipients' sera. Although BD in combination with IR results in increased amylase levels and treatment with BH4 demonstrates reduced amylase serum levels, no statistically significant differences were achieved for any group (Figure 5A). On the contrary, lipase levels in transplanted groups showed significantly higher levels, especially in BD recipients, than in sham‐operated animals (Tx BD versus Tx sham; 591.4 U/l ± 85.48 vs. 198.6 U/l ± 31.58; p < 0.01, Figure 5B). More interestingly, BH4 treatment resulted in significantly lower serum lipase levels than in nontreated BD animals (Tx BD vs. Tx BD plus BH4; 591.4 U/l ± 85.48 vs. 321.8 U/l ± 25.71; p < 0.05, Figure 5B). No significant differences in lipase levels were detected between nontransplanted groups independently of intervention or treatment.

### BD in combination with IRI leads to increased nitrosative stress

**Figure 6:**
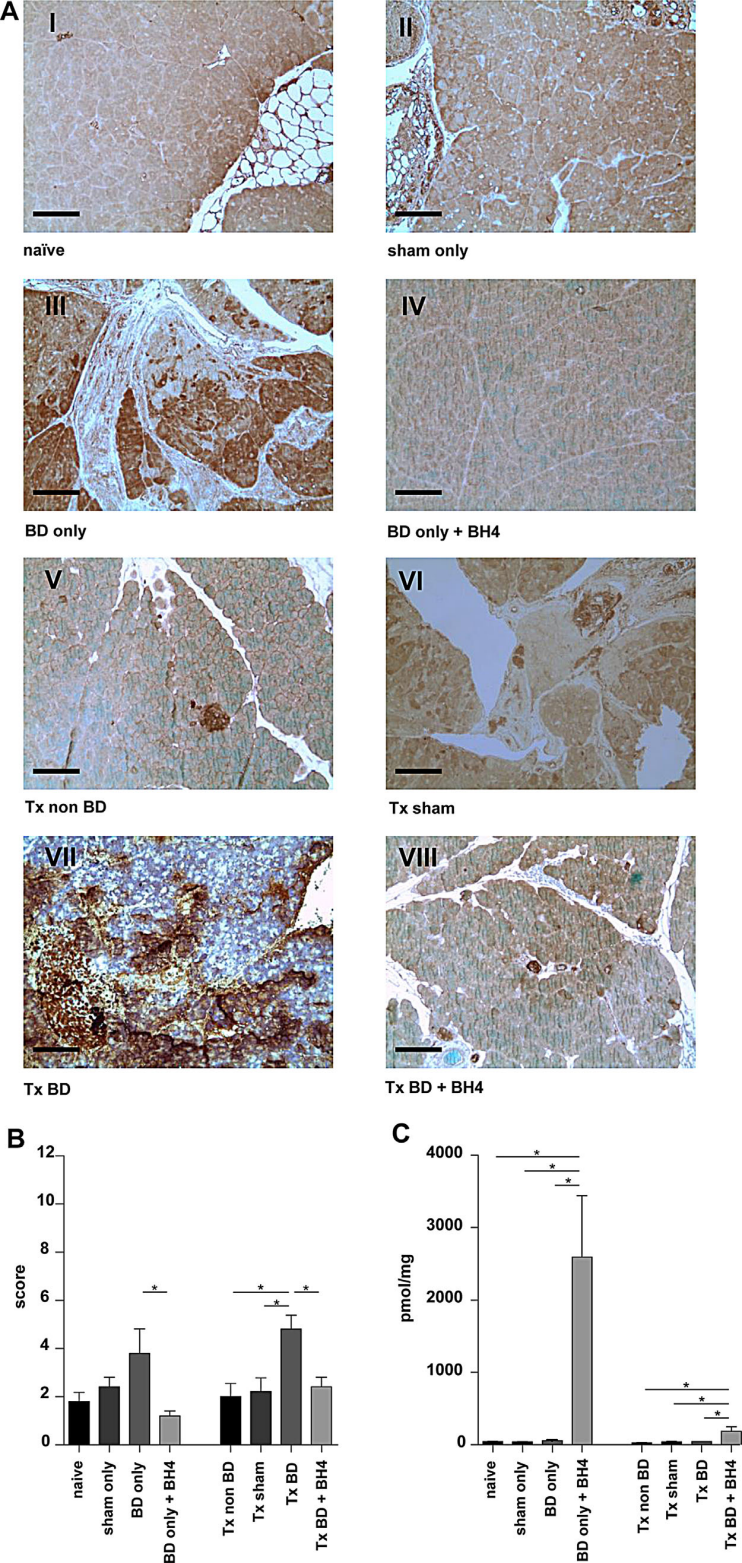
**Nitrosative stress is reduced by BH4 treatment of the BD donor** Immunohistochemical detection of nitrotyrosine in pancreatic tissue, as an indirect marker for nitrosative stress, revealed a significant impact of BD. Whereas naïve (I) and sham‐treated (II) animals showed almost no positive staining, BD donors displayed a significantly higher number of positive cells (stained dark brown), and a higher staining intensity (III). In contrast, tissue samples from BH4‐treated animals showed only few positive cells (IV). The combination of ischemia reperfusion and donor BD further aggravated these changes as the number and the staining intensity were higher (VII). Treatment with BH4 showed significant protective effects on peroxynitrite formation as the number of nitrotyrosine‐positive cells was lower (VIII). Grafts from sham‐treated (VI) and non‐BD (V) control animals displayed only a minimal number of positive cells. Representative pictures are shown for n = 5 animals/group. (B) Quantitative immunostaining score as the product of the proportion of positive cells and the staining intensity. Data are presented as mean values of n = 5 animals/group ± SEM. (C) Intragraft BH4 levels as assessed by HPLC were significantly elevated after BH4 pretreatment. Data are presented as mean values of n = 5 animals/group ± SEM and statistically significant differences were tested with one‐way ANOVA and the Tukey posttest; *p < 0.05. ANOVA, analysis of variance; BD, brain dead; BH4, tetrahydrobiopterin; HPLC, high performance liquid chromatography; SEM, standard error of the mean; Tx, transplantation.

As illustrated by representative microscopic pictures of nontransplanted pancreatic tissues, donor BD led to nitrotyrosine formation, as staining intensity and extent of positive cells were greater, albeit not significantly in the BD only group (Group III), as compared to naïve or sham animals (Groups I and II) (Figure 6A and B). Treatment with BH4 resulted in significantly reduced scores as compared to untreated animals (BD only vs. BD only plus BH4; 3.8 ± 1.0 vs. 1.2 ± 0.2; p < 0.05). However, differences in nitrotyrosine formation were more pronounced after transplantation. Mean scores in nontreated animals were significantly higher than in BH4‐treated donors (Tx BD vs. Tx BD plus BH4; 4.8 ± 0.6 vs. 2.4 ± 0.4; p < 0.05) and mean scores for the Tx non‐BD and the Tx sham group were comparable (Figure 6B).

We further detected significantly higher BH4 levels in pretreated animals at the time of organ retrieval as compared to untreated pancreata independently of BD induction (BD only vs. BD only plus BH4; 56.14 pmol/mg ± 6.04 vs. 2.595 pmol/mg ± 846.9; p < 0.05) (Figure 6B). Although dramatically reduced, BH4 levels in the treatment group were still significantly higher following 2 h of reperfusion than in nontreated groups (BD group vs. BD plus BH4; 40.46 pmol/mg ± 1.81 vs. 184. 1 pmol/mg ± 62.48; p < 0.05; Figure 6C).

### Treatment with BH4 prolongs survival of BD grafts

**Figure 7:**
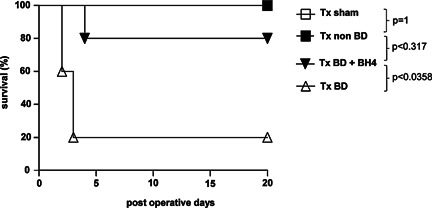
**Recipient survival is prolonged by BH4 treatment of the BD donor** Pancreata were procured from BH4 treated or untreated BD donors, respectively and transplanted into syngeneic recipients (n = 5). Grafts from sham treated and non‐BD donors respectively were transplanted into syngeneic recipients as controls. Following transplantation recipient survival was monitored for 20 days. Whereas all animals of control groups survived the entire observation period, four out of five recipients receiving a syngraft from BD donors died within 3 days posttransplantation. Treatment with BH4 resulted in significantly prolonged recipient survival after the transplantation of graft from BD donors (p < 0.05). Kaplan–Meier curve was used for survival analyses and survival differences were compared using the log rank test (*p < 0.05). BD, brain dead; BH4, tetrahydrobiopterin; Tx, transplantation.

Next, we assessed recipient survival following pancreas transplantation over an observation period of 20 days. Receiving a graft from BD donors without treatment resulted in 80% lethality due to the development of acute pancreatitis 20, 21, with four of five recipient animals dying within 48–72 h after transplantation. In contrast, BH4 treatment of BD donors resulted in significantly prolonged recipient survival in four out of five animals, one animal dying on day 4 after transplantation (Tx BD vs. Tx BD plus BH4; p < 0.05). Recipients receiving grafts from non‐BD donors and recipients receiving grafts from sham treated controls survived the entire observation period of 20 days (Figure 7).

## Discussion

While it has been well described that BD causes severe alterations in donor organs, data on the impact of donor BD on pancreatic allograft quality remain scarce 26, 27. Meanwhile, one decade ago, only two experimental studies demonstrated that BD results in pathophysiological alterations in the pancreas, reflected by microcirculatory deterioration, elevated inflammatory tissue response and histological pancreas damage reducing islet yields and functionality 28, 29. In order to address the question whether donor BD accelerates IRI, we combined the experimental model of donor BD induction and a model of pancreas transplantation in the mouse.

Microcirculation is a characteristic parameter of organ function and viability as already demonstrated by Anelescu and colleagues, who showed that microcirculatory derangements after reperfusion in the kidney correlate with impaired graft function after transplantation 30. Indeed, combining the deleterious effects of BD and IRI, microcirculation assessed by IVFM was dramatically impaired when organs from BD donors were transplanted and reperfused for 2 h as compared to naïve or sham control animals (Figure 2B and C). Contrary to already published data 27, we detected an influence of donor BD itself as compared only with naïve animals (Group III vs. Group I, p < 0.05) but not sham controls (Figure 2B).

The timely and accurate assessment of islet quality is not only important in improving isolation methods, but also critical in predicting the success of subsequent allotransplantation 31. Consequently, the method of vital stain combination applying SYTO®16 in combination with propidium iodide is widely used for assessing cell viability 32, 33. With it, cell viability showed a significant decrease in biopsies taken from BD pancreata before and after reperfusion. Contrarily, treatment using BH4 resulted in a significant higher percentage of viable cells in the transplanted group but not in the BD only group (Figure 3A and B). Our study further illustrates that the combination of donor BD and IRI resulted in significant differences essentially in the categories acinar necrosis, interstitial edema, and hemorrhage/fat necrosis and compared to sham‐treated animals (Figure 4A–E). The low scores observed in all experiment groups for inflammatory infiltrates are in line with previous observations made by our working group and might be attributed to the short reperfusion period of 2 h 20. We hypothesize that the minimal reduced cell viability and histology observed for naïve pancreata can be attributed to the damage from organ perfusion, procurement, and the staining process 34, 35, 36.

Whereas IRI aggravation from donor BD is obvious, pretreatment of the BD donor with BH4 illustrated a tremendous influence on allograft quality (Figures 2, 3, 4). This observation was also made for the detection of serum amylase and lipase. These markers are widely accepted as clinical markers indicating acinar cell necrosis and subsequent tissue damage in pancreatic organs as the increase in both parameters indicates acute pancreatitis, for example, after transplantation 37, 38. However, only serum lipase was significantly elevated following 2 h of reperfusion, showing a significant difference between BH4‐treated and nontreated grafts derived from BD donors (Figure 5B). We assume that the chosen time was too early to detect differences in terms of serum amylase, which is in line with observations showing indirect correlation between amylase levels in the serum and microcirculatory parameters 3 days following reperfusion 39. Moreover, the greater sensitivity and specificity of lipase over amylase make the former preferable for the diagnosis of pancreatitis in the clinical setting, as lipase remains unaltered in some nonpancreatic conditions whereas an increase in amylase may indicate macroamylasemia, parotitis, and some carcinomas 40, 41.

The nitration of protein tyrosine residues causes nitrotyrosine to form and thus may be considered an indirect marker for peroxynitrite formation in the context of oxidative stress 15, 18. Peroxynitrite is generated by the interaction of superoxide anions with NO and has been associated with deleterious effects on cellular and tissue function including increased oxidative reactions, lipid peroxidation, and reduction of plasma antioxidants 42, 43. Donor BD revealed a clear increase in peroxynitrite formation reflected by nitrotyrosine staining as compared with non‐BD controls. BH4 supplementation resulted in decreased nitrotyrosine immunostaining, in non and transplanted groups, indicating that BH4 prevents the tyrosine‐nitrating properties of peroxynitrite following BD and IRI, thus leading to overall reduced injury (Figure 6B). No significant depletion of BH4 was observed in our study, because 2 h after reperfusion quantification of BH4 levels in grafts derived from non‐BD, BD, and sham‐treated animals were comparable (Figure 6C). This observation might be attributed to the short cold ischemia time of approximately 45 min chosen in our experiment set‐up. However, these results clearly demonstrate that BH4 given i.m. 2 min prior to organ procurement is detectable within the pancreatic tissue 2 h after reperfusion resulting in the prevention of graft pancreatitis and prolonged recipient survival (Figure 7).

Although a comprehensive analysis of cytokine expression in pancreatic tissues is still lacking, a recent study of human pancreatic tissues identified highly upregulated mRNA levels of TNFα in pancreatic tissue from BD donors, whereas IL‐1ß or IFNy mRNA did not appear to be increased 27. In contrast, a significant induction for inflammatory cytokines including IL‐1ß and IL‐6 and adhesion molecules such as VCAM‐1 and P‐selectin was observed after BD induction in our study, corroborating the finding that donor BD itself leads to an inflammatory response in organs 23, 44, 45 (Figure 1). For instance, enhanced expression of VCAM‐1 in human pancreatic cancers has suggested a role of this marker in tumor pathogenesis, and enhanced P‐selectin expression has been attributed to acute pancreatitis 46, 47, 48.

In conclusion, we demonstrated the deleterious effects of BD on pancreatic grafts, in combination with ischemia reperfusion, which may increase the risk for graft pancreatitis and elevated immunogenicity with its negative effects on pancreas transplantation outcome. However, the most prominent finding of this study is the superiority of donor pretreatment with the essential NOS cofactor BH4 in protecting pancreatic grafts from IRI throughout all performed analyses.

## Disclosure

The authors of this manuscript have no conflicts of interest to disclose as described by the *American Journal of Transplantation*.

## Supporting information


**Table S1**: Primer for real‐time RT‐PCR.

**Figure S1**: **Intra‐arterial blood pressure following BD induction.** Hemodynamics were stable over the 180 min observation time. Although BD only (white triangle) animals as well as BD + BH4 animals (black triangle) showed a slightly lower mean arterial blood pressure than did the sham group (white square), differences between experiment groups did not reach statistical significance (n = 5 animals/group).
