# Supplementary material for: Treatment With Tetrahydrobiopterin Overcomes Brain Death–Associated Injury in a Murine Model of Pancreas Transplantation
Source: Am J Transplant. 2015 Jun 23;15(11):2865–76. doi: 10.1111/ajt.13364 (PMC4744967; doi:10.1111/ajt.13364)
Supplement: Supplementary file 1 — Table S1: Primer for real‐time RT‐PCR. [file AJT-15-2865-s001.doc]

**Oberhuber et al. Supplemental Material**

**Treatment with Tetrahydrobiopterin Overcomes Brain Death-Associated Injury in a Murine Model of Pancreas Transplantation**

***Real-time RT-PCR***

Total RNA from snap-frozen pancreatic tissues was extracted using the RNAII Kit (Macherey-Nagel; Düren, Germany) according to the manufacturer's instructions. Real-time reverse transcription polymerase chain reaction (RT-PCR) for gene expression analysis was performed with the ABI PRISM 7500 Sequence Detection System (Life Technologies, Darmstadt, Germany). Primers for IL-1ß and IL-6 were designed using Primer Express Software (Life Technologies) and validated (Table S1). Primer for VCAM-1 (Mm01320970-m1) and P-selectin (Mn01295931_m1) were purchased as Assays on Demand (Life Technologies). The PCR reaction was performed in a final volume of 25 µl containing 1 µl cDNA, 12.5 µl Master Mix (Life Technologies), 1 µl fluorogenic hybridization probe, 6µl primer mix, and 5.5 µl distilled water. Amplification consisted of a two-step PCR (40 cycles; 15s denaturation step at 95°C and 1 min annealing/extension step at 60°C). Mean Ct was calculated from double determinations, and samples were considered negative if Ct exceeded 40 cycles. Specific gene expression was normalized to the housekeeping gene hypoxanthine-guanine phosphoribosyltransferase (HPRT) given by the formula 2^-ΔCt^.

***Serum amylase and lipase***

Blood samples were assessed at the Central Institute of Medical and Chemical Laboratory Diagnostics, Innsbruck Medical University. For quantitative pancreatic amylase determination the enzymatic in-vitro test P-AMYL (No. 11555812, Cobas®, Vienna, Austria) and for lipase determination the enzymatic in vitro assay LIP (No 11821733, Cobas®, Vienna, Austria) for Roche® automated clinical chemistry analyzers were used.

***BH4 tissue levels***

To determine intrapancreatic BH4 concentrations, tissue was homogenized on ice with an ultraturrax in distilled water containing 5 mM dithioerythrol, centrifuged at 12.000 g at 4^◦^C for 10 min and then subjected to oxidation in acid or base, by a method modified from Fukushima and Nixon (1). For this purpose, 20 µl containing 0.5 M HCl and 0.05 M iodine for acidic or 20 µl 0.5 M NaOH plus 0.05 M iodine for basic oxidation were added to 100 µl supernatant. After incubation for 1 h in the dark at room temperature, 20 µl 1 M HCl was added to the basic oxidation only. Samples were then centrifuged for 2 min at 12.000 g and 4^◦^C, and 20µl 0.1 M ascorbic acid was added to reduce excess iodine. Biopterin concentrations were determined by HPLC using 10 µl injection volume, a Nucleosil 10 SA column (250 mm long, 4 mm i.d., Macherey–Nagl, Düren, Germany), eluted with 1.5 ml/min 50 mM potassium phosphate buffer, pH 3.0 and fluorescence detection (excitation 350 nm, emission 440 nm). BH4 concentrations were calculated as difference in results from oxidation in acid and base, respectively (1).

***Histopathology***

Grafts were fixed in 10% formaldehyde for 24 h, embedded in paraffin and stained with hematoxylin and eosin (H&E). For quantification purposes the semiquantitative Schmidt score was adopted, which quantifies four categories of parenchymal damage: interstitial edema, acinar necrosis, hemorrhage and fat necrosis, and inflammatory infiltrates. In each category scores ranging from 0 to 4 were assigned (2).

***Immunohistochemistry***

Tissue sections (4 µm) were cut from paraffin blocks, mounted on slips and paraffin was removed by heating in citrate buffer, pH 6.0. Endogenous peroxidase was blocked with 0.3% hydrogen peroxide. IHC for nitrotyrosine was then performed in a diaminobenzidine-tetrahydrochloride (DAB) autostainer (DAKO, Copenhagen, Denmark) using an anti-nitrotyrosine rat polyclonal antibody from Upstate Biotechnology (Lake Placid, NY, USA) at a 1:100 dilution. For staining purposes, a secondary antibody peroxidase-labeled polymer and 3,3′ DAB were used. Haemalaun was used for counterstaining and quantification was performed as described elsewhere (3). Analysis was performed in a blinded manner by an experienced pathologist.

1. Fukushima T, Nixon JC. Analysis of reduced forms of biopterin in biological tissues and fluids. Anal Biochem 1980;102: 176-188

2. Schmidt J, Rattner DW, Lewandrowski K, Compton CC, Mandavilli U, Knoefel WT, et al. A better model of acute pancreatitis for evaluating therapy. Ann Surg 1992;215: 44-56

3. Maglione M, Hermann M, Hengster P, Schneeberger S, Mark W, Obrist P, Wet al. Tetrahydrobiopterin attenuates microvascular reperfusion injury following murine pancreas transplantation. Am J Transplant 2006;6: 1551-1559

**Table S1.** Primer for real-time RT-PCR

| **Gene** | **Primer sequences** | |
| --- | --- | --- |
| **HPRT** | **sense**  **antisense**  **probe** | **5´ ATC ATT ATG CCG AGG ATT TGG AA3´**  **5´ TTG AGC ACA CAG AGG GCC A** **3´**  **5´ TGG ACA GGA CTG AAA GAC TTG CTC GAG ATG 3´** |
| **IL-1ß** | **sense**  **antisense**  **probe** | **5´ TAC AGG CTC CGA GAT GAA CAA 3´**  **5´ ATG GAG AAT ATC ACT TGT TGG TT 3´**  **5´ ACC CAT GTG AGC TGA AAG CTC TCC ACC 3´** |
| **IL-6** | **sense**  **antisense**  **probe** | **5’ TCC AGA AAC CGC TAT GAA GTT CC 3’**  **5’ GTC ACC AGC ATC AGT CCC AAG 3’**  **5’ CTC TGC AAG AGA CTT CCA TCC AGT TGC CT3’** |
