# Supplementary material for: Treatment With Tetrahydrobiopterin Overcomes Brain Death–Associated Injury in a Murine Model of Pancreas Transplantation
Source: Am J Transplant. 2015 Jun 23;15(11):2865–76. doi: 10.1111/ajt.13364 (PMC4744967; doi:10.1111/ajt.13364)
Supplement: Supplementary file 2 — Figure S1: Intra‐arterial blood pressure following BD induction. Hemodynamics were stable over the 180 min observation time. Although BD only (white triangle) animals as well as BD + BH4 animals (black triangle) showed a slightly lower mean arterial blood pressure than did the sham group (white square), differences between experiment groups did not reach statistical significance (n = 5 animals/group). [file AJT-15-2865-s002.pdf]

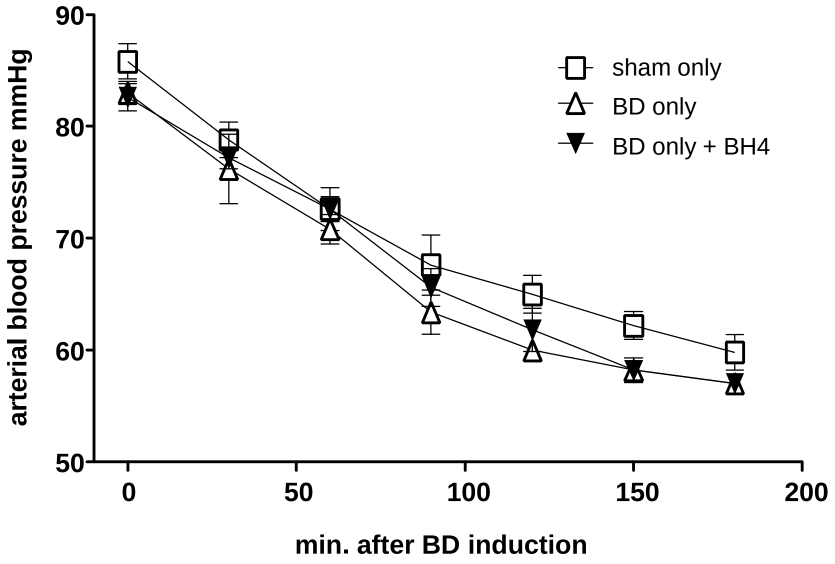

Supplemental Figure 1. Intra-arterial blood pressure following BD induction. Hemodynamics were stable over the 180 min observation time. Although BD only (white triangle) animals as well as BD + BH4 animals (black triangle) showed a slightly lower mean arterial blood pressure than did the sham group (white square), differences between experiment groups did not reach statistical significance (n=5 animals/group).
